# Comparative Effectiveness of Antipsychotics in Patients With Schizophrenia Spectrum Disorder

**DOI:** 10.1001/jamanetworkopen.2024.38358

**Published:** 2024-10-09

**Authors:** Aleksi Hamina, Heidi Taipale, Johannes Lieslehto, Markku Lähteenvuo, Antti Tanskanen, Ellenor Mittendorfer-Rutz, Jari Tiihonen

**Affiliations:** 1 Department of Forensic Psychiatry, University of Eastern Finland, Niuvanniemi Hospital, Kuopio, Finland; 2 Norwegian Centre for Addiction Research, Institute of Clinical Medicine, University of Oslo, Oslo, Norway; 3 Department of Clinical Neuroscience, Karolinska Institutet, Stockholm, Sweden

## Abstract

**Importance:**

Antipsychotics are the cornerstone of maintenance treatment in schizophrenia spectrum disorders, but it is unclear which agents should be prioritized by prescribers.

**Objective:**

To investigate the clinical effectiveness of antipsychotics, including recent market entries, in comparison with oral olanzapine in relapse and treatment failure prevention among individuals with schizophrenia spectrum disorder.

**Design, Setting, and Participants:**

This comparative effectiveness research study with a within-individual analysis included data from Swedish health care registers of inpatient and specialized outpatient care, sickness absence, and disability pensions among all individuals aged 16 to 65 years who were diagnosed with schizophrenia spectrum disorder from January 1, 2006, to December 31, 2021, including an incident cohort and a prevalent cohort.

**Exposures:**

Specific antipsychotics.

**Main Outcomes and Measures:**

The risks for psychosis relapse hospitalization and treatment failure (psychiatric hospitalization, death, or change in an antipsychotic medication) were adjusted for the temporal order of treatments, time since cohort entry, and concomitant drugs. Comparisons of all antipsychotics with oral olanzapine, the most commonly used antipsychotic, were investigated.

**Results:**

Among the full cohort of 131 476 individuals, the mean (SD) age of the study cohort was 45.7 (16.2) years (70 054 men [53.3%]). During a median follow-up of 12.0 years [IQR, 5.2-16.0 years], 48.5% of patients (N = 63 730) experienced relapse and 71.1% (N = 93 464) underwent treatment failure at least once. Compared with oral olanzapine, paliperidone 3-month long-acting injectable (LAI) was associated with the lowest adjusted hazard ratio (AHR) in the prevention of relapses (AHR, 0.66; 95% CI, 0.51-0.86), followed by aripiprazole LAI (AHR, 0.77 [95% CI, 0.70-0.84]), olanzapine LAI (AHR, 0.79 [95% CI, 0.73-0.86]), and clozapine (AHR, 0.82 [95% CI, 0.79-0.86]). Quetiapine was associated with the highest risk of relapse (AHR, 1.44 [95% CI, 1.38-1.51]). For prevention of treatment failure, paliperidone 3-month LAI was associated with the lowest AHR (AHR, 0.36 [95% CI, 0.31-0.42]), followed by aripiprazole LAI (AHR, 0.60 [95% CI, 0.57-0.63]), olanzapine LAI (AHR, 0.67 [95% CI, 0.63-0.72]), and paliperidone 1-month LAI (AHR, 0.71 [95% CI, 0.68-0.74]).

**Conclusions and Relevance:**

This comparative effectiveness research study demonstrated large differences in the risk of relapse and treatment failure among specific antipsychotic treatments. The findings contradict the widely held conception that all antipsychotics are equally effective in relapse prevention.

## Key Points

**Question:**

Do antipsychotics differ in their effectiveness to prevent relapse and treatment failure in patients with schizophrenia spectrum disorders?

**Findings:**

In this comparative effectiveness research study including Swedish health care register data for 131 476 individuals, specific antipsychotics were compared with oral olanzapine. Paliperidone 3-month long-acting injectable (LAI), aripiprazole LAI, olanzapine LAI, and clozapine were associated with the lowest risks for relapse, whereas the lowest risks for treatment failure included the first 3 agents and paliperidone 1-month LAI; in contrast, quetiapine was associated with the highest risk of relapse.

**Meaning:**

These findings contradict the notion that all antipsychotics are equally effective in relapse prevention among patients with schizophrenia spectrum disorders.

## Teaser

This comparative effectiveness research study examines whether different antipsychotics differ in their effectiveness compared with oral olanzapine to prevent relapse and treatment failure in patients with schizophrenia spectrum disorders.

## Introduction

Antipsychotic medication is the cornerstone of maintenance treatment for schizophrenia, with 3 as the number needed to treat to prevent 1 psychotic relapse.^1,2^ In schizophrenia, a relapse carries a significant risk of harm to self and others, in addition to a decrease in quality of life and social and economic consequences both for the individual and society.^1,3,4^ Aside from clozapine, however, randomized clinical trials (RCTs) have not distinguished large differences in relapse prevention among antipsychotics, and disagreement exists as to which agents, if any, should be prioritized.^5,6,7^ Antipsychotic agents may also differ in terms of safety and rates of discontinuation,^8,9^ in addition to other important outcomes that are rarely studied with trial design, including reducing mortality.

There are also other important limitations in data stemming from trials on antipsychotics. Approximately 80% of individuals with schizophrenia spectrum disorders who use antipsychotics would be ineligible for RCTs, most frequently due to somatic comorbidities, use of other psychotropic drugs, or history of suicidality or substance abuse.^10^ Thus, unsurprisingly, those individuals who would not be eligible for RCTs have worse outcomes compared with those who would be. In addition, RCTs rarely use head-to-head comparisons of several pharmaceuticals or lack statistical power for rarer outcomes, inhibiting clinical decision-making. The studies are often short in duration, making measurement of long-term outcomes difficult. Many have thus argued for the use of observational research to complement trial data.^7,11^

Previous studies have found clozapine and long-acting injectable (LAI) antipsychotics to be especially effective in preventing relapses in patients with schizophrenia.^12,13^ These previous studies, which used data from nationwide registers ending in 2013 and 2014, lack data on newly marketed antipsychotics and data on outcomes related to discontinuation or other measures of treatment failure. As population-level data start to accumulate only after market entry, collecting enough observational data to evaluate clinical effectiveness takes time and must therefore be preceded by clinical use. Moreover, because these previous studies with fewer data and with a relative lack of statistical power have used nonuse of antipsychotics as the reference, differences among specific antipsychotic agents have remained unclear. This information is of the utmost importance so that clinicians can assess which agents to prioritize. These previous studies have also only included patients with a diagnosis of schizophrenia or schizoaffective disorder, diagnoses that many patients with first-episode psychoses may not receive, thus reducing the representativeness of the patient population as a whole. In this study, we compared the effectiveness of antipsychotics for relapse and treatment failure prevention compared with oral olanzapine, the most frequently used antipsychotic, among individuals with schizophrenia spectrum disorders using national health registers in Sweden from January 1, 2006, to December 31, 2021.

## Methods

We conducted a population-based comparative effectiveness research study with a within-individual design by linking data from several Swedish health and population registers. We first identified all individuals aged 16 to 65 years diagnosed with schizophrenia spectrum disorder in Sweden during 2006 to 2021. Using a pseudonymized personal identification number, these data were then linked to national registries, which included the Swedish Prescribed Drug Register, consisting of data on all filled prescriptions from Swedish pharmacies; the National Patient Register, consisting of data on inpatient stays and diagnoses; and the Micro-Data for Analyses of the Social Insurance register, which includes sickness absences and disability pensions.

This study was approved by the Regional Ethical Review Board of Karolinska Institutet. According to current Swedish regulations, the use of registry data for research purposes does not require informed consent from individuals held in these registries. The study followed the International Society for Pharmacoeconomics and Outcomes Research (ISPOR) reporting guideline.

### Study Population

Individuals with schizophrenia spectrum disorder were identified with an *International Statistical Classification of Diseases, Tenth Revision* (*ICD-10*) diagnosis of F20-29 from inpatient and specialized outpatient care and sickness absence and disability pensions. Cohort entry was defined as the date of first diagnosis for those diagnosed from January 1, 2006, to December 31, 2021, and as January 1, 2006, for those diagnosed before. All included individuals were required to be aged 16 to 65 years at the time of the diagnosis.

We further formed an incident cohort of those who received their first-time diagnosis from January 1, 2006, to December 31, 2021; that is, individuals without a previous diagnosis of schizophrenia spectrum disorder before January 1, 2006, in either of the 2 registers (the National Patient Register and the Micro-Data for Analyses of the Social Insurance) used for the cohort formation and no antipsychotic use 15 months before the first diagnosis but allowing initiations within 3 months before diagnosis (ie, a 1-year washout period 15 to 3 months before the first diagnosis). All other individuals with a diagnosis of schizophrenia spectrum disorder were categorized as the prevalent cohort.

### Exposure

We determined antipsychotic use from the national Prescribed Drug Register, which includes all filled prescriptions from all Swedish community pharmacies since July 2005. Pharmacy dispensing records are registered according to the World Health Organization’s Anatomical Therapeutic Chemical classification, in which antipsychotics correspond to the code N05A (excluding lithium N05AN01). Only outpatient antipsychotic use was considered, as the register does not include data on drugs used in hospitals. We modeled drug-use periods (ie, estimates of when drug use started and ended) from dispensing records according to a previously described method.^14^ Antipsychotics used by the study cohort were further divided according to drug formulation into oral antipsychotics and LAIs. Concomitant use periods of 2 or more antipsychotics, regardless of length of use, were categorized as polytherapy and analyzed as a separate category.

### Outcome

The outcome of relapse was defined as hospitalization due to psychosis (*ICD-10* codes F20-F29) and treatment failure as a composite outcome of any of the following: psychiatric hospitalization (*ICD-10* codes F00-F99), death, or any change in antipsychotic medication (switch, discontinuation, or add-on of other antipsychotics). Treatment failure was only measured for monotherapies due to the inherently greater chance of outcomes in polytherapy. Data on death were extracted from the National Cause of Death Register, and psychiatric hospitalizations were from the National Patient Register, defined as an inpatient stay in an acute psychiatric ward or other psychiatric ward for at least 24 hours.

### Covariates

As we used a within-individual design, in which all time-invariant factors were automatically controlled for by the design, the analyses were only adjusted for time-dependent covariates. These covariates were time since cohort entry, temporal order of the specific antipsychotics used, and use of other psychotropic drugs (antidepressants, benzodiazepines, lithium, mood stabilizers, attention-deficit/hyperactivity disorder drugs, or drugs for addictive disorders). Comorbid conditions were identified from the National Patient Register, and drug use was from the Prescribed Drug Register. Covariate definitions are shown in eTable 1 in Supplement 1.

### Statistical Analysis

In the study’s within-individual analysis, both outcomes were treated as recurring events and analyzed with stratified Cox proportional hazards regression models.^12,15^ Each individual and their antipsychotic use and nonuse periods formed their own stratum, and follow-up time was reset to 0 after each outcome event. The analyses were conducted by comparing specific antipsychotic use in monotherapy with oral olanzapine, as that was the most frequently used antipsychotic in the study population.

As a post hoc sensitivity analysis, we tested how much low-dose use of quetiapine, which is common for treatment indications other than psychosis, contributed to the risk of relapse. This was done by removing quetiapine-use periods corresponding to 0.4 or less defined daily doses (160 mg) per day from the within-individual analysis in comparison with oral olanzapine use.

Additionally, we investigated the risk of relapse associated with specific LAIs compared head-to-head with their corresponding oral formulation in within-individual models (eg, olanzapine LAI vs oral olanzapine). To extract an aggregate result of all LAIs compared with their oral counterparts, the resulting head-to-head estimates were then meta-analyzed together with a fixed-effects model.

We further investigated the risk of relapse in traditional between-individual Cox proportional hazards regression models. In addition to the aforementioned time-dependent covariates, these analyses were adjusted for age, sex, duration of first hospital care due to psychosis, the number of relapses at cohort entry, prior use of clozapine, prior use of LAIs, prior use of lithium, previous suicide attempt, and diagnosis of substance use disorder (eTable 1 in Supplement 1).

We used the Benjamini-Hochberg method to reduce the false discovery rate, considering 2-sided *P* values <.05 as statistically significant. Original *P* values are presented in eTables 2 and 4-7 in Supplement 1. Data management and main analyses were analyzed with SAS, version 9.4 (SAS Institute Inc), and the meta-analysis of comparing LAIs with their oral counterparts was analyzed with package metafor in R, version 3.0-2 (R Project for Statistical Computing).

## Results

The full study population included 131 476 individuals with a schizophrenia spectrum disorder diagnosis (Table). The mean (SD) age was 45.7 (16.2) years, 70 054 (53.3%) were men, and 61 422 (46.7%) were women. The median follow-up was 12.0 years (IQR, 5.2-16.0 years). Olanzapine, risperidone, and aripiprazole were the most frequently used oral antipsychotics, and zuclopenthixol LAI, perphenazine LAI, and risperidone LAI were the most frequent LAIs (eTable 2 in Supplement 1), while aripiprazole, quetiapine, and risperidone were the most frequently used antipsychotics among patients who were ever-users of olanzapine (eTable 3 in Supplement 1). Similarly, olanzapine, aripiprazole, and risperidone were the most common oral antipsychotics in the incident cohort, but aripiprazole LAI, paliperidone 1-month LAI, and risperidone LAI were the most used LAIs (eTable 4 in Supplement 1).

**Table.** Characteristics of the Full Study Population and the Prevalent and Incident Subcohorts^a^

Characteristic	Full cohort (N = 131 476)	Incident cohort (n = 39 359)	Prevalent cohort (n = 92 117)	Female participants (n = 61 422)	Male participants (n = 70 054)
Age at cohort entry, mean (SD), y	45.7 (16.2)	35.8 (13.5)	49.9 (15.4)	48.5 (16.5)	43.2 (15.5)
Age group, y					
16-24	15 248 (11.6)	10 010 (25.4)	5238 (5.7)	5676 (9.2)	9572 (13.7)
25-34	21 681 (16.5)	10 756 (27.3)	10 925 (11.9)	8091 (13.2)	13 590 (19.4)
35-44	25 337 (19.3)	7580 (19.3)	17 757 (19.3)	11 002 (17.9)	14 335 (20.5)
45-54	27 820 (21.2)	6113 (15.5)	21 707 (23.6)	13 206 (21.5)	14 614 (20.9)
55-64	25 084 (19.1)	4492 (11.4)	20 592 (22.4)	13 229 (21.5)	11 855 (16.9)
≥65	16 306 (12.4)	408 (1.1)	15 898 (17.3)	10 218 (16.6)	6088 (8.7)
Sex					
Female	61 422 (46.7)	16 160 (41.1)	45 262 (49.1)	61 422 (100)	0
Male	70 054 (53.3)	23 199 (58.9)	46 855 (50.9)	0	70 054 (100)
Register source for study population identification^b^					
Inpatient care	82 404 (62.7)	14 618 (37.1)	67 786 (73.6)	40 503 (65.9)	41 901 (59.8)
Specialized outpatient care	36 219 (27.6)	20 638 (52.4)	15 581 (16.9)	15 265 (24.9)	20 954 (29.9)
Sickness absence	4856 (3.7)	2911 (7.4)	1945 (2.1)	2209 (3.6)	2647 (3.8)
Disability pension	7997 (6.1)	1192 (3.1)	6805 (7.4)	3445 (5.6)	4552 (6.5)
Previous suicide attempt	13 274 (10.1)	4412 (11.2)	8862 (9.6)	6603 (10.8)	6671 (9.5)
Substance use disorder	27 654 (21.0)	11 035 (28.0)	16 619 (18.0)	8604 (14.0)	19 050 (27.2)
Follow-up time, median (IQR), y	12.0 (5.2-16.0)	6.6 (2.8-10.7)	16.0 (7.8-16.0)	12.9 (6.0-16.0)	11.2 (4.7-16.0)
Experienced a psychotic relapse	63 730 (48.5)	25 865 (65.7)	37 865 (41.1)	28 360 (46.2)	35 370 (50.5)
Experienced a treatment failure	93 464 (71.1)	24 990 (63.5)	68 474 (74.3)	44 648 (72.7)	48 816 (69.7)

^a^ Data are presented as No. (%) unless otherwise indicated.

^b^ Register sources included the Swedish Prescribed Drug Register, consisting of data on all filled prescriptions from Swedish pharmacies; the National Patient Register, consisting of data on inpatient stays and diagnoses; and Micro-Data for Analyses of the Social Insurance register.

### Relapse

During the follow-up, 63 730 individuals (48.5%) in the cohort experienced a relapse at least once. Compared with oral olanzapine, the best-ranking antipsychotics were paliperidone 3-month LAI (adjusted hazard ratio [AHR], 0.66 [95% CI, 0.51-0.86]), aripiprazole LAI (AHR, 0.77 [95% CI, 0.70-0.84]), olanzapine LAI (AHR, 0.79 [95% CI, 0.73-0.86]), and clozapine (AHR, 0.82 [95% CI, 0.79-0.86]), followed by other LAIs, including paliperidone 1-month LAI (AHR, 0.90 [95% CI, 0.84-0.96]). Quetiapine was associated with the highest risk of relapse (AHR, 1.44 [95% CI, 1.38-1.51]). In the post hoc analysis removing small-dose use, the risk of relapse associated with quetiapine was lower but still remarkably high (AHR, 1.29 [95% CI, 1.23-1.36]). Of newer antipsychotics, cariprazine was not associated with a similar risk of relapse as oral olanzapine; however, its 95% CI was wide (AHR, 0.97 [95% CI, 0.68-1.36]). All antipsychotics were associated with a decreased risk of relapse when compared with nonuse of antipsychotics (eFigure 1A in Supplement 1). Compared with oral olanzapine, the results were similar between men and women, but there was a statistically nonsignificant trend for a higher risk of relapse among women for paliperidone 3-month and 1-month LAIs (eTables 5 and 6 in Supplement 1).

**Figure 1.**
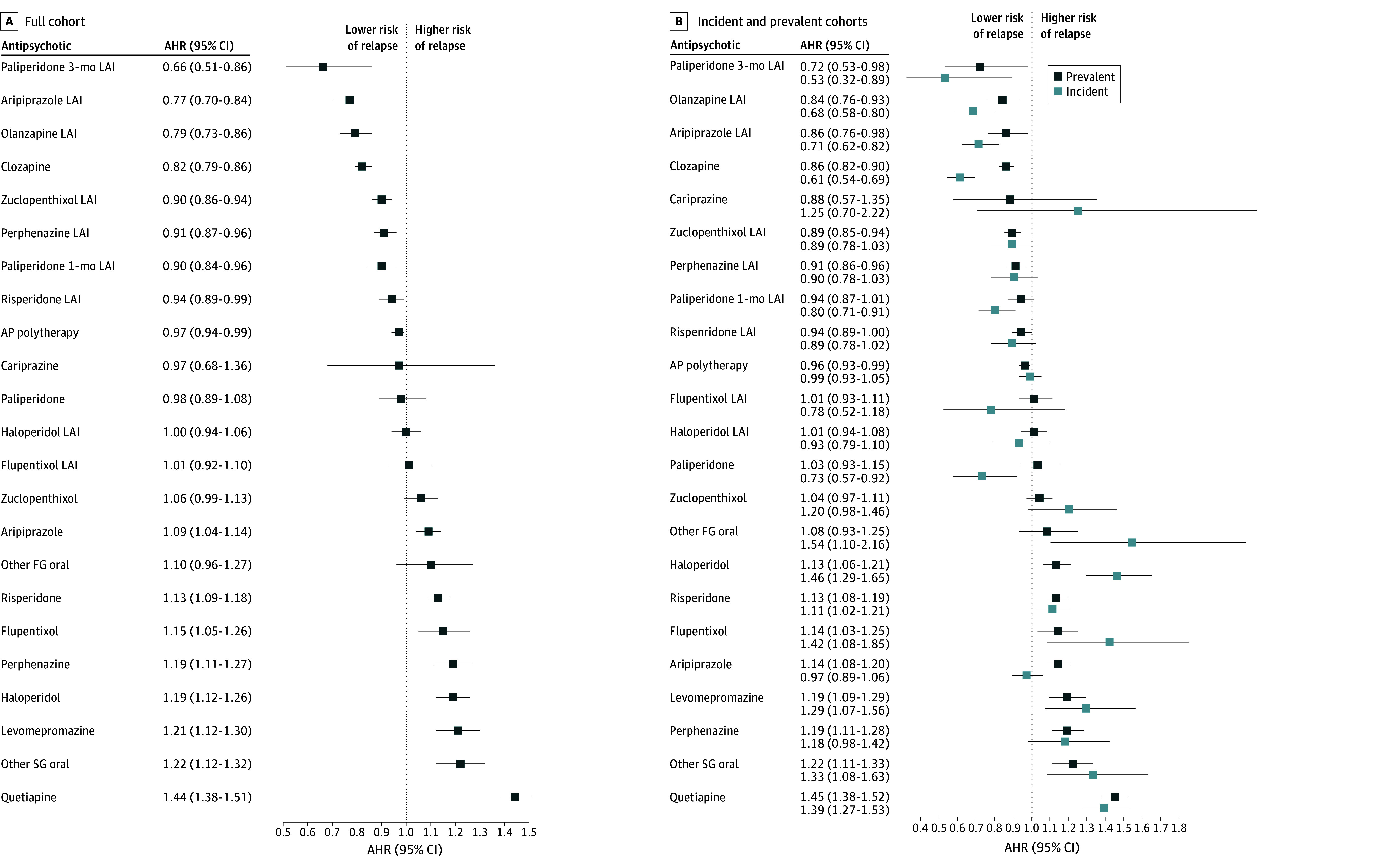
Risk of Relapse for Antipsychotics (APs) Compared With Oral Olanzapine in Within-Individual Models Comparative effectiveness of adjusted hazard ratios (AHRs) in the (A) full cohort and (B) incident and prevalent cohorts. FG indicates first generation; LAI, long-acting injectable; and SG, second generation.

The incident cohort included 39 359 individuals, of whom 25 865 (65.7% of the subcohort) experienced a relapse at least once during a median follow-up of 6.6 years (IQR, 2.8-10.7 years) (Table and eTable 4 in Supplement 1). The prevalent cohort included 92 117 individuals, of whom 37 865 (41.1%) experienced a relapse during a median follow-up of 16.0 years (IQR, 7.8-16.0 years). Overall, compared with oral olanzapine, antipsychotic effectiveness was generally similar across the 2 subcohorts, but clozapine, oral paliperidone, and oral aripiprazole showed significantly lower AHRs among the incident cohort compared with the prevalent cohort (Figure 1B). Compared with antipsychotic nonuse, olanzapine LAI, clozapine, oral paliperidone, and oral aripiprazole had lower AHRs in the incident cohort compared with the prevalent cohort (eFigure 1B in Supplement 1).

### Treatment Failure

**Figure 2.**
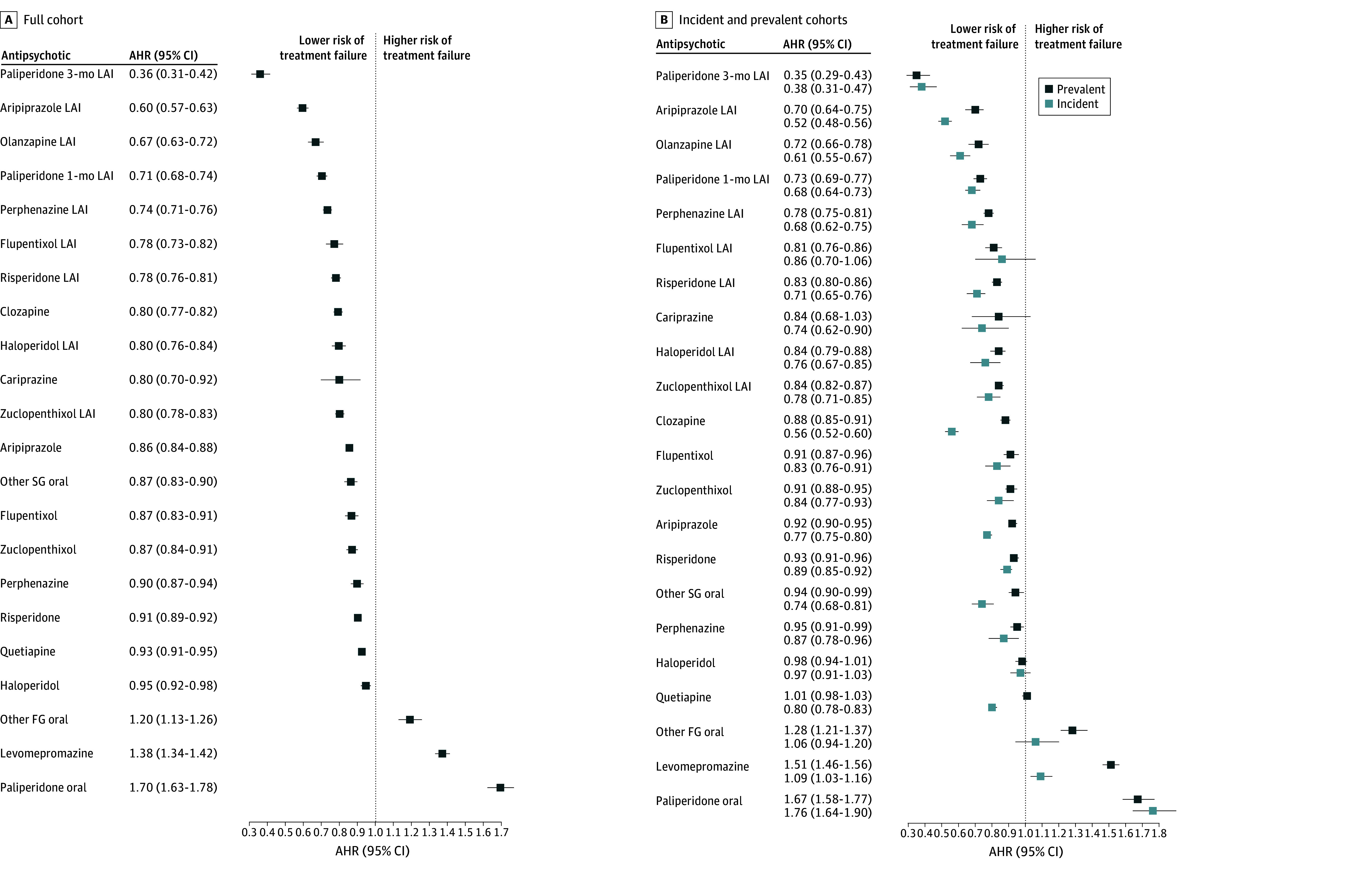
Risk of Treatment Failure for Antipsychotics Compared With Oral Olanzapine in Within-Individual Models Comparative effectiveness of adjusted hazard ratios (AHRs) in the (A) full cohort and (B) incident and prevalent cohorts. FG indicates first generation; LAI, long-acting injectable; and SG, second generation.

During a median follow-up of 12.0 years (IQR, 5.2-16.0 years), 93 464 individuals (71.1%) experienced treatment failure at least once. In within-individual models, paliperidone 3-month LAI (AHR, 0.36 [95% CI, 0.31-0.42]), aripiprazole LAI (AHR, 0.60 [95% CI, 0.57-0.63]), olanzapine LAI (AHR, 0.67 [95% CI, 0.63-0.72]), and paliperidone 1-month LAI (AHR, 0.71 [95% CI, 0.68-0.74]) were associated with the lowest risk for the outcome compared with oral olanzapine (Figure 2A). The results were largely similar between men and women; however, zuclopenthixol was associated with a higher risk of treatment failure among women compared with men (eTable 6 in Supplement 1).

In the incident cohort, 24 990 individuals (63.5%) experienced treatment failure; in the prevalent cohort, 68 474 individuals (74.3%) experienced treatment failure (Table). For this outcome, several antipsychotics were more effective in the incident cohort compared with the prevalent cohort: oral and LAI aripiprazole, clozapine, risperidone LAI, other second-generation oral antipsychotics, quetiapine, and levomepromazine (Figure 2B).

### Additional Analysis

When compared directly with their oral counterparts, LAIs were collectively associated with a lower risk of relapse (meta-analyzed: AHR, 0.81 [95% CI, 0.79-0.84]) (eFigure 1A in Supplement 1). Paliperidone 3-month LAI (AHR, 0.67 [95% CI, 0.51-0.90]), aripiprazole LAI (AHR, 0.74 [95% CI, 0.67-0.82]), and perphenazine LAI (AHR, 0.74 [95% CI, 0.68-0.80]) ranked best against their oral counterparts, and paliperidone 3-month LAI had the longest administration interval in our study. Altogether, LAIs were associated with better prevention of relapses in the incident cohort (any LAI: AHR, 0.74 [95% CI, 0.70-0.80]) compared with the prevalent cohort (any LAI: AHR, 0.84 [95% CI, 0.81-0.86]) (eFigure 2B in Supplement 1). Long-acting injectables were used by 15 746 individuals (17.1%) in the prevalent cohort and 5275 individuals (13.4%) in the incident cohort.

In the traditional between-individual models on the outcome of relapse, the rank order of antipsychotics compared with olanzapine use was similar to that of the within-individual models (eTable 7 in Supplement 1). However, only paliperidone 3-month LAI (AHR, 0.73 [95% CI, 0.56-0.96]), aripiprazole LAI (AHR, 0.79 [95% CI, 0.72-0.86]), olanzapine LAI (AHR, 0.91 [95% CI, 0.84-0.99]), and paliperidone 1-month LAI (ARH, 0.93 [95% CI, 0.87-1.00]) were associated with a decreased risk of relapse compared with oral olanzapine.

## Discussion

In this nationwide comparative effectiveness research study with a within-individual design among patients with schizophrenia spectrum disorders, we found large differences in the risk of relapses and treatment failure among specific antipsychotics. For both outcomes, especially paliperidone 3-month LAI, aripiprazole LAI, olanzapine LAI, and clozapine fared well in comparison with the most commonly used antipsychotic, oral olanzapine. In contrast, quetiapine systematically performed worse than other antipsychotics. To our knowledge, this is the largest study to date to investigate antipsychotic effectiveness in patients with schizophrenia spectrum disorders, which enabled us to detect many statistically significant findings despite a large number of specific medications and corrections for multiple comparisons.

Similar to our study, a recent network meta-analysis of RCTs found the best-performing antipsychotics to prevent relapse in schizophrenia spectrum disorders to be aripiprazole, paliperidone, and olanzapine.^5^ As all 3 are available both as oral and LAI formulations, the authors of the meta-analysis argue that new treatments should prioritize these medicines in early schizophrenia.^5^ This is in contrast to a commonly held conception that antipsychotics are similarly effective. The authors of another recent network meta-analysis of RCTs made the argument that antipsychotics prevent relapses to a comparable degree, while the adverse effects profiles of antipsychotics differ to a greater extent.^6^ The choice of pharmacotherapy should thus accommodate the clinical scenario and the preferences of each individual patient.^6^ However, initiating treatment with an oral antipsychotic for which an LAI formulation exists may facilitate a later transfer to an LAI, which seems to be supported by our findings.

The antipsychotic with the longest administration interval in our study, paliperidone 3-month LAI, ranked best in both relapse and treatment failure. Smaller previous studies have found paliperidone 3-month LAI to be associated with fewer psychiatric hospitalizations compared with oral antipsychotics or aripiprazole LAI^16^ or compared with the time before initiating paliperidone 3-month LAI.^17^ Whether this is merely a function of the long administration interval of the drug or the drug itself remains to be seen in future studies with other LAIs with longer than a 1-month administration interval. Overall, LAIs have been found to be more effective in preventing relapses than oral antipsychotics in most observational research, especially when a within-individual design is implemented.^12,13,18^ Moreover, some of the most up-to-date meta-analyses of RCTs have found LAIs to be effective when compared against placebo but also when compared against oral antipsychotics,^5,11,19^ although the effect sizes for the latter finding are small.^11^ This is likely at least partly due to the trial effect and the strict inclusion criteria and follow-up in RCTs, all increasing adherence in comparison with patients in a clinical setting. As described in the current study, LAIs were collectively more effective in preventing relapse than their oral counterparts (AHR, 0.81 [95% CI, 0.79-0.84]). This may not be surprising, as a previous analysis from our research group found that LAI treatment was 67% less likely to be interrupted compared with oral antipsychotics.^20^ Nonadherence to pharmacological treatment continues to be a major hurdle in schizophrenia, for which LAIs are 1 efficient countermeasure.^21^

One common finding of our study and of RCT meta-analyses is the poor performance of quetiapine,^5,6^ which persisted in the post hoc sensitivity analysis removing small-dose use. Despite its poor performance, quetiapine was one of the most used antipsychotics in our cohort. As the risk for weight gain and sedation are also significant,^6^ quetiapine perhaps should not be considered a first-line maintenance treatment option in schizophrenia. Our results also suggest that clozapine, which is the criterion standard for treatment-resistant schizophrenia,^22,23,24^ performed better on relapse prevention among the incident cohort compared with the prevalent cohort. This calls into question the treatment guidelines to only start the drug in a later phase of the disorder.^23,24^ Similarly, the use of LAIs was still infrequent in the incident cohort (13.4%), yet we found olanzapine LAI to be more effective for relapse prevention among incident patients when the comparison was antipsychotic nonuse. The same was likely true of aripiprazole LAI and paliperidone 1-month LAI, although the AHR CIs of the incident and prevalent cohorts overlapped slightly (eFigure 1B in Supplement 1). These results indicate that the use of LAIs was likely more effective in preventing early relapses than later ones. Early use of LAIs is also supported by RCTs including patients with early-phase schizophrenia, in which, compared with oral antipsychotics, LAIs increase the time to relapse^25,26^ and reduce the risk of relapse.^26,27^ However, a recent systematic review of all clinical practice guidelines in the English language found a significant disparity in the recommendation of LAI uptake, underlining the contradictory stances on LAI use in early schizophrenia.^7^ This may at least in part be due to the reliance on trial data in treatment guidelines, which likely skew the recommendations to apply best to the patients who are less severely ill and most adherent to treatment.^7^

### Strengths and Limitations

The main strength of this study is the use of nationwide registers to construct a large and representative cohort of patients in the general population without strict inclusion criteria. This allowed for the analysis of patients without selection bias stemming from comorbidities, willingness to participate in trials, or socioeconomic status. The use of within-individual analyses also avoided selection bias related to fixed patient characteristics, which is commonly introduced in observational studies with between-individual analyses.

This study also has some limitations. The results of our between-individual analyses, in which the AHRs were systematically higher than in the within-individual models, suggest that such selection and consequent residual confounding were seen in these results, although the rank order of the studied antipsychotics was similar to that in the within-individual analyses. The results from within-individual analyses, however, were mainly limited to individuals with variation in the exposure and the outcome. Similarly, prior stabilization on shorter-acting LAIs before the paliperidone 3-month LAI may have inflated its results to an extent, which may have also been reflected in the poor results of oral paliperidone on the outcome of treatment failure. The analyses were adjusted for the temporal order of the antipsychotic and time since cohort entry, but it is possible that some residual confounding and/or confounding by indication remained, also due to lacking information on fluctuation, severity of underlying symptoms, and possible underreporting of some covariates, such as substance use disorders. However, it is important to note that individuals with the most difficult clinical presentation and/or nonadherence were prescribed clozapine or LAIs, and yet these antipsychotics were associated with the lowest comparative risk of relapse in this study.

## Conclusions

The findings of this comparative effectiveness research study suggest that antipsychotics, especially LAIs and clozapine, were effective in preventing relapses and treatment failure among individuals with schizophrenia spectrum disorders and even more so in individuals who were newly diagnosed. Of newly marketed pharmaceuticals, especially paliperidone 3-month LAI but also cariprazine, fared well in comparison with other second-generation antipsychotics. The findings, combined with previous results from RCTs, suggest that it may be beneficial to initiate treatment with the most effective antipsychotics that are available as both oral and LAI formulations.

## Notes

Supplement 1. **eTable 1.** Covariate Definitions **eTable 2.** Relapse and Treatment Failure Associated With Specific Antipsychotics Compared With Oral Olanzapine in Within-Individual Models **eTable 3.** Distribution of Antipsychotic Use Among Oral Olanzapine Ever-Users **eTable 4.** Risk of Relapse Associated With Specific Antipsychotics Compared With Oral Olanzapine in Stratified Analyses of the Incident and Prevalent Cohorts in Within-Individual Models **eTable 5.** Numbers of Users, Person-Years, and Outcomes Among Women and Men in the Full Cohort **eTable 6.** Sex-Stratified Risk of Relapse and Treatment Failure Associated With Specific Antipsychotics Compared With Oral Olanzapine in Within-Individual Models **eTable 7.** Risk of Relapse Associated With the Use of Specific Antipsychotics Compared With Oral Olanzapine in the Between-Individual Model **eFigure 1**. Adjusted Hazard Ratios (AHRs) for Relapse Compared With Nonuse in Within-Individual Models in (A) the Full Cohort and (B) the Incident and Prevalent Cohorts **eFigure 2.** Risk of Relapse Associated With Specific Long-Acting Injectable Antipsychotics (LAIs) Compared Head-to-Head With Their Corresponding Oral Formulation in a Within-Individual Model Among (A) the Whole Cohort and (B) Prevalent and Incident Cohorts

Supplement 2.
Data Sharing Statement
