# Supplementary material for: Comparative Effectiveness of Antipsychotics in Patients With Schizophrenia Spectrum Disorder
Source: JAMA Netw Open. 2024 Oct 9;7(10):e2438358. doi: 10.1001/jamanetworkopen.2024.38358 (PMC11465102; doi:10.1001/jamanetworkopen.2024.38358)
Supplement: Supplement 1. — eTable 1. Covariate Definitions eTable 2. Relapse and Treatment Failure Associated With Specific Antipsychotics Compared With Oral Olanzapine in Within-Individual Models eTable 3. Distribution of Antipsychotic Use Among Oral Olanzapine Ever-Users eTable 4. Risk of Relapse Associated With Specific Antipsychotics Compared With Oral Olanzapine in Stratified Analyses of the Incident and Prevalent Cohorts in Within-Individual Models eTable 5. Numbers of Users, Person-Years, and Outcomes Among Women and Men in the Full Cohort eTable 6. Sex-Stratified Risk of Relapse and Treatment Failure Associated With Specific Antipsychotics Compared With Oral Olanzapine in Within-Individual Models eTable 7. Risk of Relapse Associated With the Use of Specific Antipsychotics Compared With Oral Olanzapine in the Between-Individual Model eFigure 1. Adjusted Hazard Ratios (AHRs) for Relapse Compared With Nonuse in Within-Individual Models in (A) the Full Cohort and (B) the Incident and Prevalent Cohorts eFigure 2. Risk of Relapse Associated With Specific Long-Acting Injectable Antipsychotics (LAIs) Compared Head-to-Head With Their Corresponding Oral Formulation in a Within-Individual Model Among (A) the Whole Cohort and (B) Prevalent and Incident Cohorts [file jamanetwopen-e2438358-s001.pdf]

Supplemental Online Content

Hamina A, Taipale H, Lieslehto J, et al. Comparative effectiveness of antipsychotics for relapse and treatment failure prevention in patients with schizophrenia spectrum disorder. *JAMA Netw Open.* 2024;7(10):e2438358. doi:10.1001/jamanetworkopen.2024.38358

eTable 1. Covariate Definitions
eTable 2. Relapse and Treatment Failure Associated With Specific Antipsychotics Compared With Oral Olanzapine in Within-Individual Models
eTable 3. Distribution of Antipsychotic Use Among Oral Olanzapine Ever-Users
eTable 4. Risk of Relapse Associated With Specific Antipsychotics Compared With Oral Olanzapine in Stratified Analyses of the Incident and Prevalent Cohorts in Within-Individual Models
eTable 5. Numbers of Users, Person-Years, and Outcomes Among Women and Men in the Full Cohort
eTable 6. Sex-Stratified Risk of Relapse and Treatment Failure Associated With Specific Antipsychotics Compared With Oral Olanzapine in Within-Individual Models
eTable 7. Risk of Relapse Associated With the Use of Specific Antipsychotics Compared With Oral Olanzapine in the Between-Individual Model
eFigure 1. Adjusted Hazard Ratios (AHRs) for Relapse Compared With Nonuse in Within-Individual Models in (A) the Full Cohort and (B) the Incident and Prevalent Cohorts
eFigure 2. Risk of Relapse Associated With Specific Long-Acting Injectable Antipsychotics (LAIs) Compared Head-to-Head With Their Corresponding Oral Formulation in a Within-Individual Model Among (A) the Whole Cohort and (B) Prevalent and Incident Cohorts

This supplemental material has been provided by the authors to give readers additional information about their work.

© 2024 Hamina A et al. *JAMA Network Open*.

eTable 1. Covariate Definitions

Variable	Definition
**Covariates adjusted for in within-individual and between-individual analyses**	
Order of treatments	Order of specific antipsychotics is continuously updated in the models, categorized as 1st, 2nd, 3rd, >3rd
Time since cohort entry	Time since cohort entry is continuously updated in the models, categorized as 0–1, 1–3, >3 years
Exposure to other psychotropic medications	Continuously updated variables, drug use periods were split each time when exposure to either of the drug groups listed below changed (i.e., use started or stopped).
Antidepressants	ATC code: N06A
Benzodiazepines and related drugs	ATC codes: N05BA, N05CD, N05CF
Lithium	ATC code: N05AN01
Mood stabilizers	ATC codes: N03AG01, N03AF01, N03AX09
ADHD drugs	ATC code: N06BA
Drugs for addictive disorders	ATC codes: N07BB, N07BC
**Additional covariates adjusted for in the between-individual analyses**	
Age	Age at baseline
Sex	Registered legal sex at baseline
Duration of first hospital care due to psychosis	In days, categorized as 0, 1–13, 14–27, 28–55, 56–83, >83
Number of relapses at cohort entry	Prior ICD-10 diagnoses F20-F29, categorized as ≤1, 2-3, >3
Previous suicide attempt	Prior ICD-10 diagnoses of X60-X84, Y10-Y34, yes vs. no (continuously updated in the model)
Diagnosis of substance use disorder	Prior ICD-10 diagnoses of F10-F19, yes vs. no (continuously updated in the model)
Prior use of LAI	Any prior use of LAI products, extracted by Nordic Article Numbers, yes vs. no (continuously updated in the model)
Prior use of clozapine and/or lithium	ATC codes: N05AN01 and N05AH02, yes vs. no (continuously updated in the model)

ADHD = Attention deficit hyperactivity disorder; ATC = Anatomical-Therapeutic-Chemical; ICD-10 = international classification of diseases, version 10; LAI = long-acting injectables

© 2024 Hamina A et al. *JAMA Network Open*.

eTable 2. Risk of Relapse and Treatment Failure Associated With Specific Antipsychotics Compared With Oral Olanzapine in Within-Individual Models

					Relapse			Treatment failure		
**Antipsychotic**	**Users**	**Person-years**	**Relapses**	**Treatment Failures**	**aHR (95% CI)**	**p-value**	**BHC p-values**	**aHR (95% CI)**	**p-value**	**BHC p-values**
Paliperidone LAI-3M	102	414	650	2809	0.66 (0.51-0.86)	0.0017	0.0029	0.36 (0.31-0.42)	<.0001	<.0001
Aripiprazole LAI	805	2421	3451	10397	0.77 (0.70-0.84)	<.0001	<.001	0.60 (0.57-0.63)	<.0001	<.0001
Olanzapine LAI	732	1579	2351	2076	0.79 (0.73-0.86)	<.0001	<.001	0.67 (0.63-0.72)	<.0001	<.0001
Clozapine	10563	7476	42483	4956	0.82 (0.79-0.86)	<.0001	<.001	0.80 (0.77-0.82)	<.0001	<.0001
Zuclopenthixol LAI	5955	5693	19501	3222	0.90 (0.86-0.94)	<.0001	<.001	0.81 (0.78-0.83)	<.0001	<.0001
Perphenazine LAI	4679	4635	16394	160252	0.91 (0.87-0.96)	0.0004	0.0008	0.90 (0.87-0.94)	<.0001	<.0001
Paliperidone LAI-1M	1693	3380	5624	28330	0.90 (0.84-0.96)	0.0013	0.0024	0.71 (0.68-0.74)	<.0001	<.0001
Risperidone LAI	2998	4381	10803	4631	0.94 (0.89-0.99)	0.0144	0.0203	0.78 (0.76-0.81)	<.0001	<.0001
AP Polytherapy	64561	55765	171803	12291	0.97 (0.94-0.99)	0.0102	0.0153	NA	NA	NA
Cariprazine	62	460	230	14194	0.97 (0.68-1.36)	0.8433	0.92	0.80 (0.70-0.92)	0.0017	0.0018
Paliperidone oral	826	2934	2328	4737	0.98 (0.89-1.08)	0.6535	0.7469	1.70 (1.63-1.78)	<.0001	<.0001
Haloperidol LAI	2832	2741	9106	11982	1.00 (0.94-1.06)	0.9801	0.9801	0.80 (0.76-0.84)	<.0001	<.0001
Olanzapine	18849	42714	114930	77565	Reference			Reference		
Flupentixol LAI	1346	1344	5384	2847	1.01 (0.92-1.10)	0.8856	0.9241	0.78 (0.73-0.82)	<.0001	<.0001
Zuclopenthixol	3469	5599	18791	5356	1.06 (0.99-1.13)	0.0744	0.0992	0.87 (0.84-0.91)	<.0001	<.0001
Aripiprazole	6006	18804	36987	6226	1.09 (1.04-1.14)	0.0002	0.0004	0.86 (0.84-0.88)	<.0001	<.0001
Other FG oral	446	2863	3885	33512	1.10 (0.96-1.27)	0.1559	0.1902	1.20 (1.13-1.26)	<.0001	<.0001
Risperidone	9915	23977	70969	373	1.13 (1.09-1.18)	<.0001	<.001	0.91 (0.89-0.93)	<.0001	<.0001
Flupentixol	1613	4107	15935	5771	1.15 (1.05-1.26)	0.0024	0.0038	0.87 (0.84-0.91)	<.0001	<.0001
Perphenazine	2439	4494	13066	294	1.19 (1.11-1.27)	<.0001	<.001	0.74 (0.71-0.76)	<.0001	<.0001
Haloperidol	4105	9216	21995	8727	1.19 (1.12-1.26)	<.0001	<.001	0.95 (0.92-0.98)	0.0007	0.0008
Levomepromazine	1681	7962	11284	14132	1.21 (1.12-1.30)	<.0001	<.001	1.38 (1.34-1.42)	<.0001	<.0001
Other SG oral	1591	3638	8994	5356	1.22 (1.12-1.32)	<.0001	<.001	0.87 (0.83-0.90)	<.0001	<.0001
Quetiapine	6640	19790	37386	33512	1.44 (1.38-1.51)	<.0001	<.001	0.93 (0.91-0.95)	<.0001	<.0001

aHR = adjusted hazard ratio; AP = antipsychotic; BHC = Benjamini-Hocherberg corrected; FG = first generation; LAI = long-acting injectable; NA = not applicable; SG = second generation

© 2024 Hamina A et al. *JAMA Network Open*.

eTable 3. Distribution of Antipsychotic Use Among Oral Olanzapine Ever-Users (N = 42,714)

	N of users	%
Levomepromazine	3321	7.8
Perphenazine	1471	3.4
Perphenazine LAI	1627	3.8
Haloperidol	3349	7.8
Haloperidol LAI	913	2.1
Flupentixol	1406	3.3
Flupentixol LAI	395	0.9
Zuclopenthixol	1925	4.5
Zuclopenthixol LAI	1988	4.7
Clozapine	2127	5.0
Olanzapine	42,714	100.0
Olanzapine LAI	1264	3.0
Quetiapine	9318	21.8
Risperidone	8346	19.5
Risperidone LAI	1805	4.2
Aripiprazole	9795	22.9
Aripiprazole LAI	1311	3.1
Paliperidone LAI-1M	1732	4.1
Paliperidone LAI-3M	193	0.5
AP Polytherapy	25,897	60.6
Other LAI	45	0.1
Cariprazine	262	0.6
Paliperidone oral	1631	3.8
Other SG oral	1711	4.0
Other FG oral	1102	2.6

AP = antipsychotic; FG = first generation, LAI = long-acting injectable; SG = second generation

© 2024 Hamina A et al. *JAMA Network Open*.

eTable 4. Risk of Relapse Associated With Specific Antipsychotics Compared With Oral Olanzapine in Stratified Analyses of the Incident and Prevalent Cohorts in Within-Individual Models

	Incident cohort						Prevalent cohort					
**Antipsychotic**	**Events**	**Users**	**Person-years**	**aHR (95%CI)**	**p-value**	**BHC p-values**	**Events**	**Users**	**Person-years**	**aHR (95%CI)**	**p-value**	**BHC p-values**
Paliperidone LAI-3M	33	186	261.73	0.53 (0.32-0.89)	0.0158	0.0369	71	233	401.66	0.72 (0.53-0.98)	0.0346	0.0531
Clozapine	743	1254	3196.46	0.61 (0.54-0.69)	<.0001	0.0004	9820	6222	39286.23	0.86 (0.82-0.90)	<.0001	0.0003
Olanzapine LAI	261	811	968.93	0.68 (0.58-0.80)	<.0001	0.0004	537	829	1463.59	0.84 (0.76-0.93)	0.0010	0.0021
Aripiprazole LAI	438	1450	2047.8	0.71 (0.62-0.82)	<.0001	0.0004	410	1038	1512.65	0.86 (0.76-0.98)	0.0253	0.0416
Paliperidone oral	124	1039	484.97	0.73 (0.57-0.92)	0.0073	0.0218	702	1895	1842.83	1.03 (0.93-1.15)	0.5772	0.6322
Flupentixol LAI	44	140	197.72	0.78 (0.52-1.18)	0.2421	0.3032	1304	1206	5199.33	1.01 (0.93-1.11)	0.7674	0.8023
Paliperidone LAI-1M	463	1425	1770.07	0.80 (0.71-0.91)	0.0007	0.027	1247	1985	3867.2	0.94 (0.87-1.01)	0.1067	0.1444
Risperidone LAI	415	1053	1462.92	0.89 (0.78-1.02)	0.0974	0.1467	2658	3416	9469.59	0.94 (0.89-1.00)	0.0373	0.0536
Zuclopenthixol LAI	387	789	994.06	0.89 (0.78-1.03)	0.1082	0.1467	5593	4923	18538.68	0.89 (0.85-0.94)	<.0001	0.0003
Perphenazine LAI	424	790	1243.92	0.90 (0.78-1.03)	0.1130	0.1467	4268	3853	15176.18	0.91 (0.86-0.96)	0.0004	0.0009
Haloperidol LAI	316	561	765.48	0.93 (0.79-1.10)	0.4058	0.492	2575	2214	8406.35	1.01 (0.94-1.08)	0.8375	0.8375
Aripiprazole	1742	8706	12321.73	0.97 (0.89-1.06)	0.4658	0.5127	4264	10098	24665.29	1.14 (1.08-1.20)	<.0001	0.0003
AP Polytherapy	6831	14907	17875.15	0.99 (0.93-1.05)	0.6218	0.647	57428	40472	153445.2	0.96 (0.93-0.99)	0.0134	0.0237
Olanzapine	4476	18100	24454.91	Reference			14373	24614	90474.77	Reference		
Risperidone	1736	7761	10533.61	1.11 (1.02-1.21)	0.0153	0.0369	8179	16216	60435.19	1.13 (1.08-1.19)	<.0001	0.0003
Perphenazine	211	710	793.36	1.18 (0.98-1.42)	0.0887	0.144	2228	3784	12272.93	1.19 (1.11-1.28)	<.0001	0.0003
Zuclopenthixol	202	839	873.64	1.20 (0.98-1.46)	0.0719	0.12	3267	4760	17917.79	1.04 (0.97-1.11)	0.2347	0.2841
Cariprazine	26	246	116.82	1.25 (0.70-2.22)	0.4545	0.5127	36	214	113.01	0.88 (0.57-1.35)	0.5477	0.6299
Levomepromazine	210	1803	1084.03	1.29 (1.07-1.56)	0.0089	0.0218	1471	6159	10200.14	1.19 (1.09-1.29)	<.0001	0.0003
Other SG oral	231	953	1030.38	1.33 (1.08-1.63)	0.0073	0.0218	1360	2685	7963.75	1.22 (1.11-1.33)	<.0001	0.0003
Quetiapine	1486	7181	10136.43	1.39 (1.27-1.53)	<.0001	0.0023	5154	12609	27249.07	1.45 (1.38-1.52)	<.0001	0.0003
Flupentixol	135	780	1008.93	1.42 (1.08-1.85)	0.0108	0.0248	1478	3327	14925.97	1.14 (1.03-1.25)	0.0098	0.0188
Haloperidol	642	2480	2211.66	1.46 (1.29-1.65)	<.0001	0.0012	3463	6736	19783.74	1.13 (1.06-1.21)	0.0002	0.0005
Other FG oral	68	519	228.44	1.54 (1.10-2.16)	0.0128	0.0218	378	2344	3656.18	1.08 (0.93-1.25)	0.3181	0.3817

aHR = adjusted hazard ratio; AP = antipsychotic; BHC = Benjamini-Hocherberg corrected; FG = first generation, LAI = long-acting injectable; SG = second generation

© 2024 Hamina A et al. *JAMA Network Open*.

eTable 5. Numbers of Users, Person-Years, and Outcomes Among Women and Men in the Full Cohort

	Women				Men			
	**Users**	**Person-years**	**Relapse events**	**Treatment failure events**	**Users**	**Person-years**	**Relapse events**	**Treatment failure events**
Levomepromazine	3861	6007.78	832	6310	4101	5276.4	849	5981
Perphenazine	2541	7889.41	1459	3632	1953	5176.88	980	2594
Perphenazine LAI	2313	8582.16	2220	5448	2322	7811.76	2459	4949
Haloperidol	4763	12654.36	2191	7841	4453	9341.03	1914	6353
Haloperidol LAI	1217	4344.81	1321	2296	1524	4761.08	1511	2660
Flupentixol	2395	9719.61	964	3506	1712	6215.29	649	2265
Flupentixol LAI	739	3211.52	735	1665	605	2172.94	611	1182
Zuclopenthixol	3114	11116.34	2029	5157	2485	7675.08	1440	3570
Zuclopenthixol LAI	2696	9559.96	2694	5984	2997	9941.23	3261	5998
Clozapine	2935	16604.1	4289	5895	4541	25878.59	6274	8237
Olanzapine	18999	52849.07	8122	36366	23715	62080.62	10727	41199
Olanzapine LAI	554	813.16	238	760	1025	1537.56	494	1316
Quetiapine	10049	20794.36	3479	17871	9741	16591.14	3161	15641
Risperidone	11918	36464.49	4846	16942	12059	34504.31	5069	15572
Risperidone LAI	1911	4823.72	1262	3490	2470	5979.6	1736	4426
Aripiprazole	9311	19195.06	3031	14821	9493	17791.95	2975	13509
Aripiprazole LAI	1043	1532.31	335	1244	1378	1918.9	470	1565
Paliperidone LAI-1M	1404	2283.33	753	1953	1976	3340.73	940	2784
Paliperidone LAI-3M	149	234.35	49	106	265	415.59	53	188
AP Polytherapy	26163	76573.72	29760	NA	29602	95229.76	34801	NA
Other LAI	141	676.26	146	231	131	612.71	129	201
Cariprazine	164	71.29	23	117	296	158.54	39	256
Paliperidone oral	1360	977.72	397	2173	1574	1350.07	429	2458
Other SG oral	2145	5636.45	960	3328	1493	3357.68	631	2028
Other FG oral	1427	2139.56	220	1570	1436	1745.05	226	1652

AP = antipsychotic; FG = first generation, LAI = long-acting injectable; NA = not applicable; SG = second generation

© 2024 Hamina A et al. *JAMA Network Open*.

eTable 6. Sex-Stratified Risk of Relapse and Treatment Failure Associated With Specific Antipsychotics Compared With Oral Olanzapine in Within-Individual Models

	Relapse						Treatment failure					
	Women			Men			Women			Men		
	**aHR (95% CI)**	**p-value**		**aHR (95% CI)**	**p-value**	**BHC p-values**	**aHR (95% CI)**	**p-value**		**aHR (95% CI)**	**p-value**	**BHC p-values**
Aripiprazole LAI	0.79 (0.68-0.91)	0.001	0.0034	0.75 (0.67-0.85)	<.0001	0.0003	0.61 (0.57-0.67)	<.0001	0.0001	0.59 (0.55-0.63)	<.0001	0.0002
Paliperidone LAI-3M	0.84 (0.58-1.21)	0.3415	0.5123	0.53 (0.37-0.77)	0.0008	0.0016	0.33 (0.26-0.43)	<.0001	0.0001	0.37 (0.31-0.44)	<.0001	0.0002
Clozapine	0.84 (0.79-0.90)	<.0001	0.0006	0.80 (0.76-0.85)	<.0001	0.0003	0.82 (0.78-0.86)	<.0001	0.0001	0.78 (0.75-0.81)	<.0001	0.0002
Olanzapine LAI	0.85 (0.74-0.99)	0.0305	0.0610	0.75 (0.68-0.84)	<.0001	0.0003	0.72 (0.65-0.80)	<.0001	0.0001	0.64 (0.59-0.69)	<.0001	0.0002
Zuclopenthixol LAI	0.89 (0.84-0.96)	0.0013	0.0039	0.90 (0.85-0.96)	0.0007	0.0015	0.82 (0.78-0.85)	<.0001	0.0001	0.80 (0.76-0.83)	<.0001	0.0002
Perphenazine LAI	0.92 (0.85-0.99)	0.018	0.0006	0.91 (0.85-0.98)	0.0096	0.0118	0.71 (0.68-0.75)	<.0001	0.0001	0.77 (0.73-0.81)	<.0001	0.0002
Cariprazine	0.92 (0.54-1.59)	0.7769	0.8475	0.99 (0.63-1.55)	0.9673	0.9673	0.85 (0.66-1.08)	0.1807	0.1886	0.78 (0.66-0.92)	0.0026	0.0033
Risperidone LAI	0.92 (0.85-1.00)	0.0461	0.0010	0.95 (0.88-1.02)	0.1322	0.0003	0.78 (0.74-0.82)	<.0001	0.0001	0.79 (0.75-0.82)	<.0001	0.0002
Paliperidone oral	0.96 (0.84-1.11)	0.6029	0.8039	0.99 (0.87-1.14)	0.913	0.9527	1.75 (1.64-1.86)	<.0001	0.0001	1.66 (1.57-1.76)	<.0001	0.0002
Paliperidone LAI-1M	0.98 (0.89-1.09)	0.715	0.8171	0.83 (0.76-0.91)	<.0001	0.0003	0.76 (0.71-0.81)	<.0001	0.0001	0.67 (0.64-0.71)	<.0001	0.0002
Olanzapine	Reference			Reference			Reference			Reference		
AP Polytherapy	1.00 (0.96-1.05)	0.8331	0.8603	0.93 (0.90-0.97)	0.0001	0.0003	NA	NA		NA	NA	
Flupentixol LAI	1.01 (0.90-1.14)	0.8603	0.8603	1.01 (0.89-1.15)	0.8447	0.9215	0.76 (0.70-0.83)	<.0001	0.0001	0.81 (0.74-0.88)	<.0001	0.0002
Zuclopenthixol	1.03 (0.94-1.12)	0.571	0.8039	1.11 (1.01-1.22)	0.0259	0.0366	0.83 (0.79-0.87)	<.0001	0.0001	0.95 (0.90-1.00)	0.0543	0.0592
Other FG oral	1.05 (0.86-1.27)	0.6445	0.8092	1.18 (0.98-1.43)	0.0885	0.1180	1.13 (1.04-1.22)	0.0027	0.0029	1.28 (1.18-1.38)	<.0001	0.0002
Haloperidol LAI	1.07 (0.98-1.18)	0.1205	0.1928	0.95 (0.88-1.03)	0.1976	0.2258	0.83 (0.77-0.89)	<.0001	0.0001	0.78 (0.73-0.83)	<.0001	0.0002
Aripiprazole	1.09 (1.02-1.16)	0.0132	0.0317	1.10 (1.03-1.18)	0.0027	0.0050	0.84 (0.82-0.86)	<.0001	0.0001	0.88 (0.86-0.91)	<.0001	0.0002
Risperidone	1.12 (1.06-1.19)	0.0002	0.0010	1.15 (1.09-1.22)	<.0001	0.0003	0.89 (0.86-0.92)	<.0001	0.0001	0.93 (0.90-0.96)	<.0001	0.0002
Flupentixol	1.13 (1.00-1.27)	0.0456	0.0790	1.21 (1.05-1.39)	0.0087	0.0139	0.87 (0.82-0.92)	<.0001	0.0001	0.90 (0.84-0.96)	<.0001	0.0002
Levomepromazine	1.15 (1.04-1.28)	0.008	0.0213	1.28 (1.16-1.43)	<.0001	0.0003	1.37 (1.31-1.43)	<.0001	0.0001	1.39 (1.34-1.45)	<.0001	0.0002
Haloperidol	1.18 (1.09-1.28)	<.0001	0.0006	1.20 (1.11-1.31)	<.0001	0.0003	0.92 (0.88-0.96)	<.0001	0.0001	1.00 (0.96-1.04)	0.9768	0.9768
Other SG oral	1.20 (1.08-1.34)	0.0007	0.0028	1.26 (1.10-1.43)	0.0007	0.0015	0.89 (0.84-0.93)	<.0001	0.0001	0.94 (0.88-1.00)	0.0074	0.0089
Perphenazine	1.22 (1.12-1.34)	<.0001	0.0006	1.15 (1.04-1.28)	0.0069	0.0118	0.89 (0.84-0.93)	<.0001	0.0001	0.94 (0.88-1.00)	0.0345	0.0394
Quetiapine	1.46 (1.37-1.56)	<.0001	0.0006	1.44 (1.35-1.53)	<.0001	0.0003	0.92 (0.89-0.94)	<.0001	0.0001	0.95 (0.92-0.98)	0.0002	0.0003

aHR = adjusted hazard ratio; AP = antipsychotic; BHC = Benjamini-Hocherberg corrected; FG = first generation, LAI = long-acting injectable; NA = not applicable; SG = second generation

© 2024 Hamina A et al. *JAMA Network Open*.

eTable 7. Risk of Relapse Associated With the Use of Specific Antipsychotics Compared With Oral Olanzapine in the Between-Individual Model

Antipsychotic	aHR (95% CI)	p-value	Corrected p-value
Paliperidone LAI-3M	0.73 (0.56-0.96)	0.0218	0.0291
Aripiprazole LAI	0.79 (0.72-0.86)	<.0001	0.0002
Olanzapine LAI	0.91 (0.84-0.99)	0.0281	0.0355
Paliperidone LAI-1M	0.93 (0.87-1.00)	0.0341	0.0409
Risperidone LAI	0.98 (0.93-1.03)	0.4656	0.4858
Olanzapine	Reference		
Aripiprazole	1.03 (0.99-1.07)	0.1486	0.1698
Other FG oral	1.06 (0.94-1.20)	0.3101	0.3383
Perphenazine LAI	1.08 (1.03-1.13)	0.0018	0.0025
Cariprazine	1.08 (0.82-1.41)	0.5884	0.5884
Zuclopenthixol LAI	1.11 (1.06-1.15)	<.0001	0.0002
Risperidone	1.14 (1.10-1.18)	<.0001	0.0002
Clozapine	1.16 (1.12-1.21)	<.0001	0.0002
Flupentixol LAI	1.17 (1.09-1.26)	<.0001	0.0002
Flupentixol	1.19 (1.10-1.27)	<.0001	0.0002
Other SG oral	1.19 (1.11-1.27)	<.0001	0.0002
Paliperidone oral	1.22 (1.12-1.34)	<.0001	0.0002
Levomepromazine	1.22 (1.15-1.30)	<.0001	0.0002
Haloperidol LAI	1.25 (1.18-1.32)	<.0001	0.0002
Quetiapine	1.29 (1.25-1.34)	<.0001	0.0002
AP Polytherapy	1.31 (1.28-1.34)	<.0001	0.0002
Haloperidol	1.33 (1.27-1.39)	<.0001	0.0002
Perphenazine	1.34 (1.26-1.43)	<.0001	0.0002
Zuclopenthixol	1.36 (1.29-1.43)	<.0001	0.0002
Other LAI	1.65 (1.40-1.95)	<.0001	0.0002

Adjusted for time-varying use of antidepressants, mood stabilizers, benzodiazepines and related drugs, ADHD drugs and drugs for alcohol and opioid addiction, the temporal order of antipsychotics, age, sex, duration of first hospital care due to psychosis, the number of relapses at cohort entry, prior use of clozapine, prior use of LAI, prior use of lithium, previous suicide attempt and diagnosis of substance use disorder.

© 2024 Hamina A et al. *JAMA Network Open*.

eFigure 1. Adjusted Hazard Ratios (AHRs) for Relapse Compared With Nonuse in Within-Individual Models in (A) the Full Cohort and (B) the Incident and Prevalent Cohorts

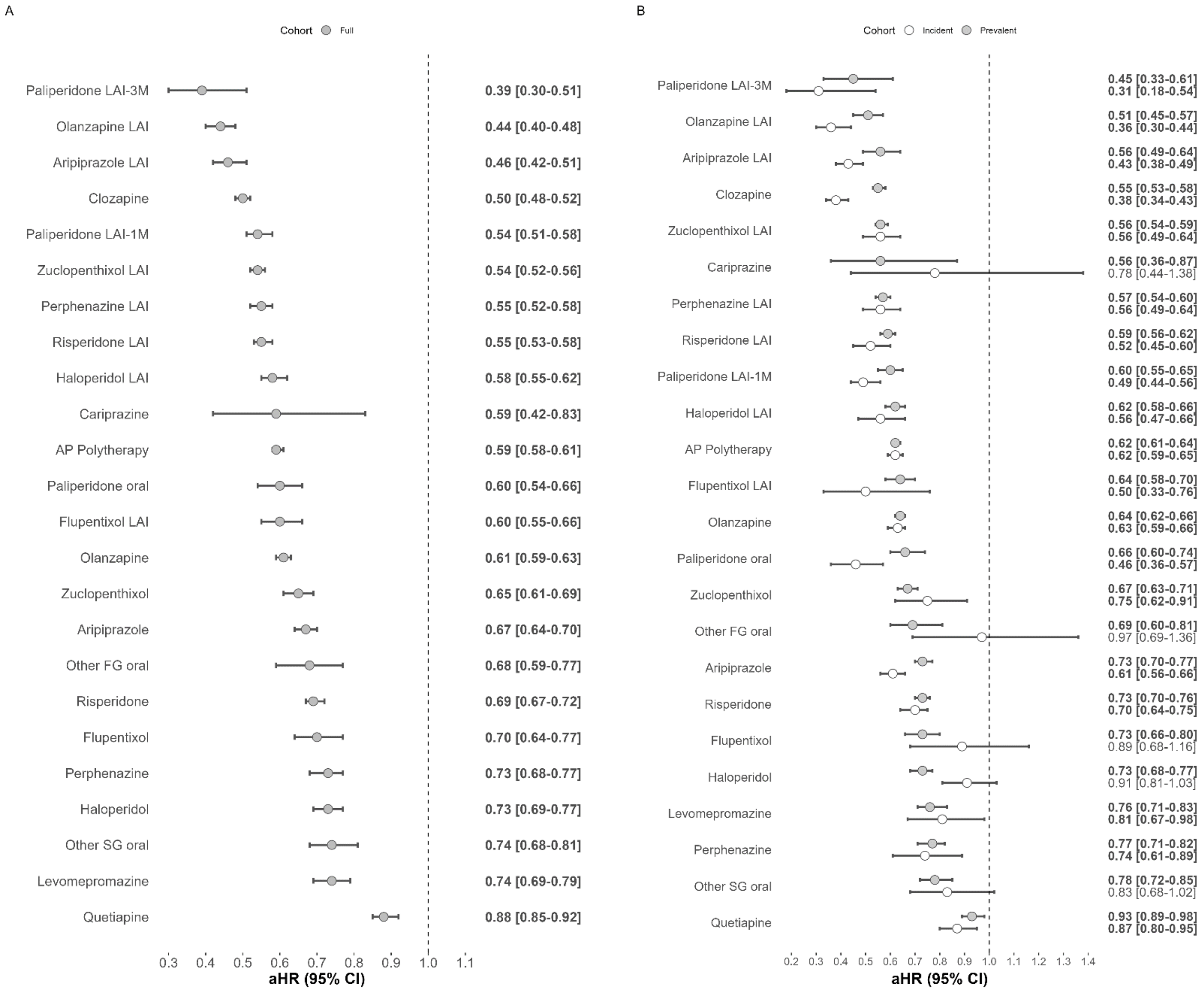

FG=first generation; LAI=long-acting injectable; SG=second generation. Benjamini–Hochberg corrected statistically significant values in bold.

© 2024 Hamina A et al. *JAMA Network Open*.

eFigure 2. Risk of Relapse Associated With Specific Long-Acting Injectable Antipsychotics (LAIs) Compared Head-to-Head With Their Corresponding Oral Formulation in a Within-Individual Model Among (A) the Whole Cohort and (B) Prevalent and Incident Cohorts

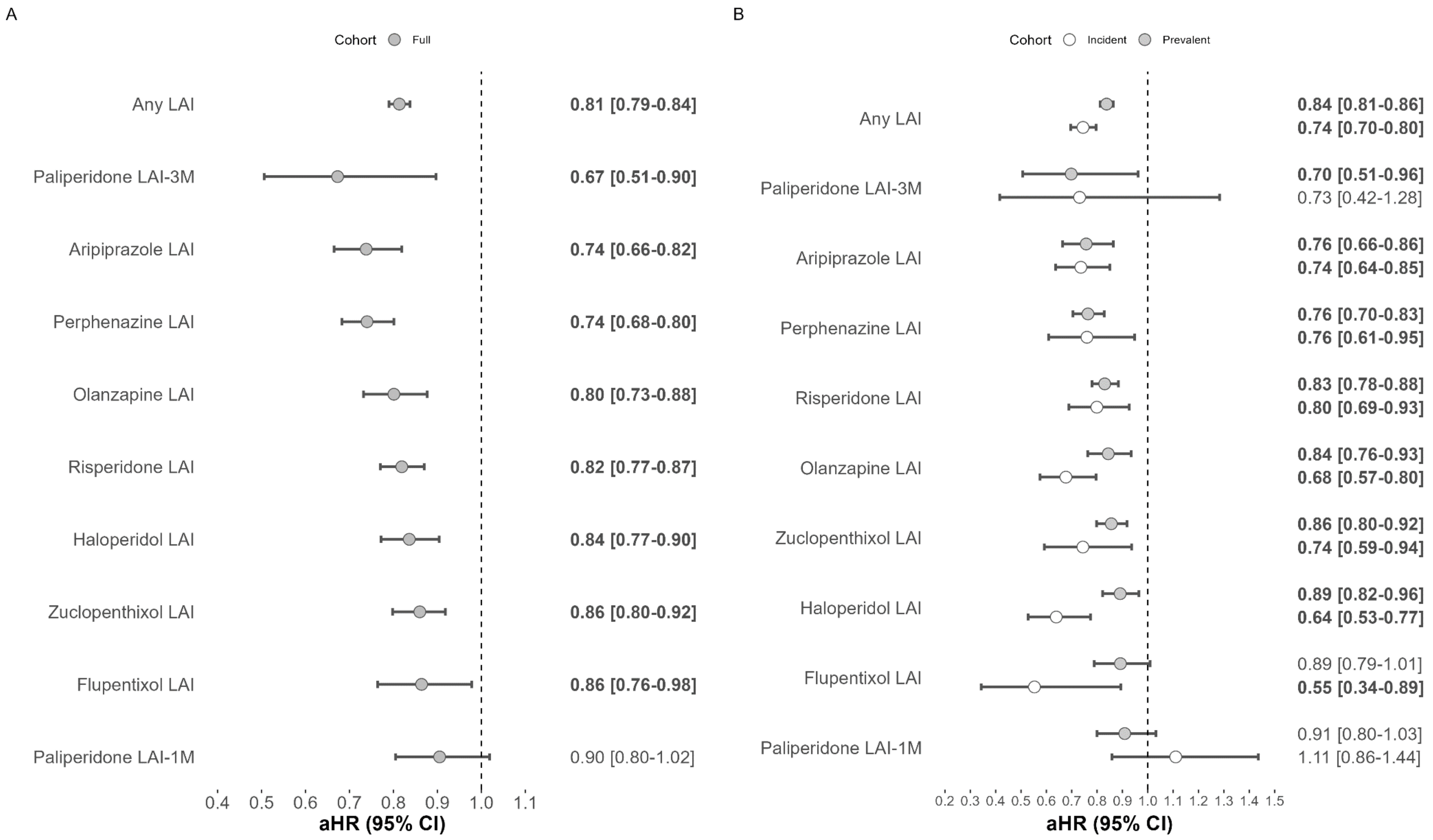

Any LAI combines all LAIs and their corresponding orals, meta-analysis result. Benjamini–Hochberg corrected statistically significant values in bold.

© 2024 Hamina A et al. *JAMA Network Open*.
